# Brain without Anatomy: Construction and Comparison of Fully Network-Driven Structural MRI Connectomes

**DOI:** 10.1371/journal.pone.0096196

**Published:** 2014-05-01

**Authors:** Olga Tymofiyeva, Etay Ziv, A. James Barkovich, Christopher P. Hess, Duan Xu

**Affiliations:** 1 Department of Radiology and Biomedical Imaging, University of California San Francisco, San Francisco, California, United States of America; 2 Department of Neurology, University of California San Francisco, San Francisco, California, United States of America; 3 Department of Pediatrics, University of California San Francisco, San Francisco, California, United States of America; Wake Forest School of Medicine, United States of America

## Abstract

MRI connectomics methods treat the brain as a network and provide new information about its organization, efficiency, and mechanisms of disruption. The most commonly used method of defining network nodes is to register the brain to a standardized anatomical atlas based on the Brodmann areas. This approach is limited by inter-subject variability and can be especially problematic in the context of brain maturation or neuroplasticity (cerebral reorganization after brain damage). In this study, we combined different image processing and network theory methods and created a novel approach that enables atlas-free construction and connection-wise comparison of diffusion MRI-based brain networks. We illustrated the proposed approach in three age groups: neonates, 6-month-old infants, and adults. First, we explored a data-driven method of determining the optimal number of equal-area nodes based on the assumption that all cortical areas of the brain are connected and, thus, no part of the brain is structurally isolated. Second, to enable a connection-wise comparison, alignment to a “reference brain” was performed in the network domain within each group using a matrix alignment algorithm with simulated annealing. The correlation coefficients after pair-wise network alignment ranged from 0.6102 to 0.6673. To test the method’s reproducibility, one subject from the 6-month-old group and one from the adult group were scanned twice, resulting in correlation coefficients of 0.7443 and 0.7037, respectively. While being less than 1 due to parcellation and noise, statistically, these values were significantly higher than inter-subject values. Rotation of the parcellation largely explained the variability. Through the abstraction from anatomy, the developed framework allows for a fully network-driven analysis of structural MRI connectomes and can be applied to subjects at any stage of development and with substantial differences in cortical anatomy.

## Introduction

Although general domains of eloquent function can be allocated to specific regions of the cerebral cortex, complex brain function requires communication between regions and effective *integration* of distributed neural activity. On a large scale, this essential task is carried out by the white matter fibers, which interconnect cortical areas and deep gray matter structures. The idea that the brain can be viewed and studied as a network has found its *in vivo* implementation with the recent emergence of “MRI connectomics,” proposed by *Hagmann* [1] and *Sporns et al.* [2] in 2005. MRI connectomics treats the brain as a network of either structural or functional connections between brain regions and provides potential new information about its efficiency and mechanisms of disruption. In this approach, patches of the cortical surface serve as *nodes* of the network, and white matter fibers that have been reconstructed using diffusion tractography or temporal correlations of the fMRI signal serve as *connections* or *edges*. Subsequently, analysis based on *graph theory* can be applied, and by means of the provided abstraction, the complexity is reduced, and the comparison of the network organization can be performed even across species [3]. In humans, this approach has been applied to studying network disruption in schizophrenia, Alzheimer’s disease, multiple sclerosis, and other neurologic and psychiatric disorders (for a recent review, see [4]).

To enable the abstract treatment of neural systems as networks, important decisions have to be made, as the location, size, and functional properties of the nodes have to be eliminated from network models of the nodes. In other words, how does one define the nodes? Usually, the brain of the studied subject is registered to a generic brain with defined Brodmann areas. Brodmann areas were originally defined using cytoarchitectural differences between brain regions. However, the borders of the areas, with a few exceptions, do not match the sulci and gyri of the cortical surface or any other external morphological features [5], [6]. The question arises regarding the accuracy of mapping those areas onto the brain in each individual case, especially in the context of brain maturation or abnormal anatomy. Cases of astounding cerebral reorganization after brain damage (neuroplasticity) have been reported [7]. To take an extreme example, in young children undergoing hemispherectomy for the treatment of intractable epilepsy, cortical plasticity and change of connectivity allow the contralateral hemisphere to assume the functions of the lost hemisphere without significant neurologic deficits [8]. It is questionable whether the registration of such a reorganized brain to a standard atlas based on the Brodmann areas will provide valuable insights into the newly adjusted brain network. Similarly, albeit not as dramatic, biases can be introduced when studying the developing brain of neonates, who have immature sulcation, if one partially or completely relies on adult brain atlases. Even in the case of normal anatomy of the adult brain, different subjects can have, for example, different dominant hemispheres. Is there a way to analyze and compare brain networks without the constraints of standardized anatomy?.

Recently, an atlas- and template-free approach to mapping brain networks was proposed, which is based on an equal-area parcellation scheme and is not constrained by anatomy [9]–[11]. Without any assumptions with respect to gyral or sulcal anatomic landmarks, this approach avoids anatomical biases and is, therefore, especially helpful in studying the developing brain. It has been utilized in the study of infants with hypoxic ischemic encephalopathy (HIE) [9], [11] and brain maturation [10]. Global network properties, such as the average clustering coefficient and characteristic path length, were shown to correlate with the neurological outcome of infants with HIE and with the maturation stage. While the approach allows for comparison of global network measures, local comparison of specific nodes and connections has not yet been possible.

*The purpose* of this study was to develop a framework for the construction and connection-wise comparison of fully network-driven structural connectomes and illustrate its application in three age groups, with four subjects in each group: neonates, 6-month-old infants, and adults. First, we explore the method of determining the optimal number of nodes for the atlas-free, equal-area parcellation scheme based on the assumption that all cortical areas of the brain are connected to other areas through some set of edges [11]. Thus, we define the optimal parcellation as the finest parcellation that still interconnects the whole brain, leaving no nodes isolated. Second, to enable edge-wise comparison, network alignment is performed within each group. The sum network is obtained and mapped to the anatomy of a “reference brain.” This method enables the comparison of both global and local network properties of any two brain networks. To estimate the method’s reproducibility, we scanned one subject from the 6-month-old group and one from the adult group twice. To estimate the variability introduced by the parcellation, we varied the position of the equal-area nodes by gradual rotation of the reference sphere.

## Materials and Methods

### Subjects

All of the MRI scans were compliant with the Health Insurance Portability and Accountability Act (HIPAA) and the study was approved by the Committee on Human Research (CHR) of the University of California, San Francisco. Written informed consent was obtained from all adult participants. In the case of neonates and infants, written informed parental consent was obtained.

The study included three age groups: 4 neonates imaged in the first 4–5 days of life, 4 six-month-old infants, and 4 adults (age 24–31 years). All subjects were female. The two pediatric groups consisted of infants with transient encephalopathy at birth, but none of the patients had clinical or imaging evidence of brain injury after the immediate neonatal period. The six-month-old infants had a normal neurological outcome assessed at the day of the MRI by pediatric neurologists. Infants with seizures were excluded from the study. All adults were right-handed.

To estimate the method’s reproducibility, one 6-month-old subject and one adult were scanned twice (with a slight head displacement).

### MRI Data Acquisition

The subjects were scanned on a 3T GE MR scanner using a spin echo (SE) echo planar imaging (EPI) diffusion tensor imaging (DTI) sequence with a FOV = 24–25.6 cm, a 128×128 matrix, slice thickness of 1.8–2 mm, 30 directions, and b = 700 s/mm^2^ for the pediatric groups and b = 1000 s/mm^2^ for adults. Lower b-values were used for imaging of the neonates due to the very high water content of these structurally immature brains. Forty-five to 66 contiguous slices were acquired through the entire brain. The scan time for the DTI sequence was approximately four minutes for the babies and nine minutes for the adults. All subjects were scanned in an eight-channel adult head coil.

**Figure 1:**
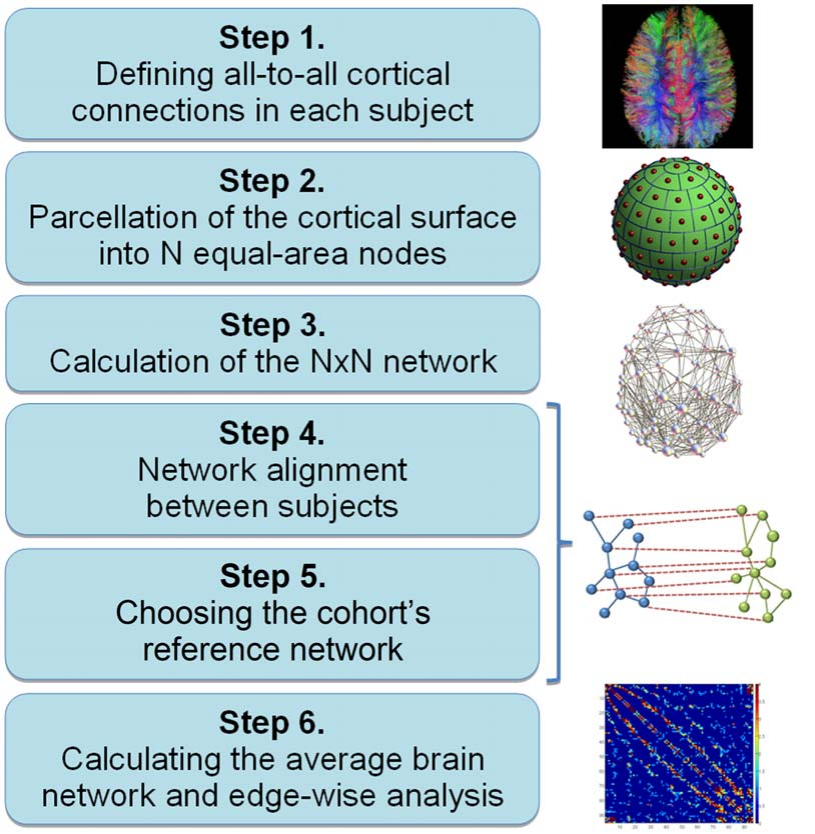
Flowchart: Construction and analysis of fully network-driven structural connectomes in any population.

The six main steps for construction and analysis of a fully network-driven structural connectome in any population are described in Figure 1. In addition to the indicated software packages, implementation of the intermediate steps was done using Matlab 7.14 (MathWorks, Natick, USA).

### Step 1. Defining all-to-all Cortical Connections

Any network can be represented as a connectivity (or adjacency) matrix, which consists of nodes and edges. In the case of a large-scale diffusion MRI-based structural connectome, reconstructed streamlines commonly represent (but do not directly correspond to) anatomical white matter connections between cortical areas. The path from diffusion-weighted images to the all-to-all connectivity matrix (“dense connectome”) has been described previously [9]–[11] and includes (*i*) data quality assurance and eddy current and motion correction [9], [12], (*ii*) diffusion tensor reconstruction and deterministic whole-brain streamline fiber tractography performed using the Diffusion Toolkit software package [13] and Fiber Assignment by Continuous Tracking (FACT) algorithm (seed density = 1 seed per voxel, threshold angle = 35°), (*iii*) extracting the subcortical surface 4–6 mm below the cortex using the diffusion images, and (*iv*) construction of the “dense connectome” by detecting which points *i* and *j* of the subcortical surface are connected by streamlines and storing the corresponding element (*i,j*) of the connectivity matrix. Prior to constructing the networks, streamline length thresholding was performed on the tractography result to account for noise in the DTI data and oversimplification of the tensor modeling. As in the previous study, the minimum length of the streamline necessary to be included in the network construction was set to 5 mm for neonates, 10 mm for 6-month-old infants, and 15 mm for adults [10].

### Step 2. Parcellation of the Cortical Surface into N Equal-area Nodes

**Figure 2:**
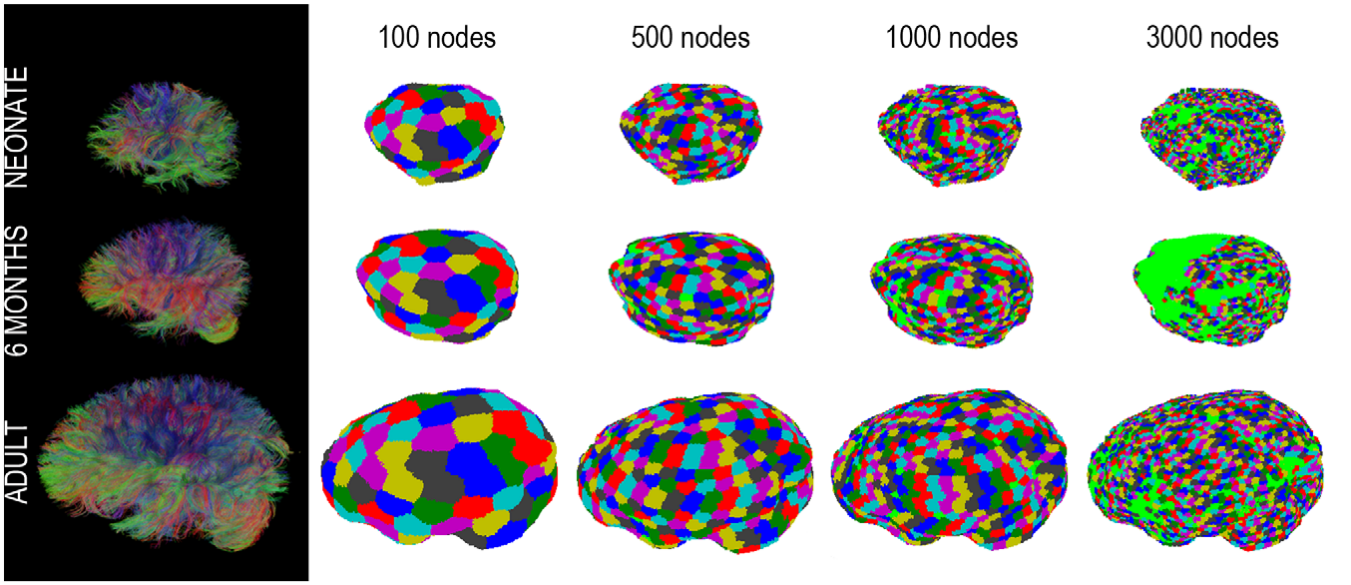
Tractograms and the giant component mapped on the brain (*N* = 100, 500, 1000, 3000) for a representative subject from each age group: neonates, 6-month-old infants, and adults. Light green areas indicate nodes of the parcellation that are not part of the giant component.

To enable analysis of macroscopic brain networks, the dense connectome needs to be downscaled by means of a definition of the brain’s large-scale regions and connections. Possible approaches include [14]: *i)* predefined anatomical templates, *ii)* randomly generated parcels of roughly equal size, and, potentially, *iii)* connectivity-based parcellations aiming to delineate large-scaled brain regions by exploiting similarities in structural and functional connectivity patterns [15]. Since the purpose of our study was to propose an alternative to atlas-based connectome analysis and since connectivity-based parcellations appear to have an inherent degree of variability [15], we chose the second approach. Automated, atlas-free parcellation of the cortical surface was based on equal-area sphere partitioning [9]. It is similar to the randomly generated parcels of roughly equal size used in some other studies [14], however, in our case parcellation was performed on each individual brain separately without co-registering them to a common anatomical template. A unit sphere was first divided into regions of equal area and the set of center points of the regions was determined, in order to serve as the node reference points. The sphere was then scaled to the brain surface and every point on the brain surface was assigned to the closest node reference point. This simple and practical approach resulted in nodes of a similar size. Based on previous results [11], the numbers of nodes were set to *N* = 10, 50, 90, 95, 100, 105, 110, 150, 300, 500, 1000, and 3000. For each parcellation, all groups of interconnected nodes (“components”) were enumerated by identifying a spanning tree of each component using a depth-first search algorithm. This allowed for the identification of the *giant component* – the largest connected component [16] (Fig. 2). Assuming all cortical areas of the brain are connected and no part of the brain is structurally isolated, the optimal parcellation was empirically determined as the finest parcellation that still interconnects the whole brain, leaving no nodes isolated.

### Step 3. Calculation of the N×N Network

Calculation of the *N×N* networks was performed by combining dense connectome entries, which resulted in some nodes being connected by a large number of streamlines. While multiple approaches to weighting network connections have been proposed, no index derived from tractography has been proposed so far that can quantify “connection strength” in a physiological or anatomical context [17]. Therefore, the resulting *N×N* networks were binarized at a threshold of 1 streamline: two nodes were considered connected if at least one connecting streamline was present.

#### Global network measures

Global network metrics of the resulting *N×N* networks were then calculated for each subject using the Brain Connectivity Toolbox developed for Matlab (BCT, http://www.brain-connectivity-toolbox.net) [18]. Because network measures depend on the number of edges, edge distribution, and the size of the connected component, we normalized the calculated measures using corresponding measures derived from random graphs with the same number of edges and the same degree distribution [19]. Various network metrics were calculated for each network, including scaled (normalized) clustering coefficient relative to a population of random networks (*Cr = C/C_rand_*), scaled characteristic path length relative to a population of random networks (*Lr = L/L_rand_*), maximized modularity (*Q*), and small-world index (*swi*), defined as a ratio (*C/C_rand_*)/(*L/L_rand_*). In the randomized networks, each edge was rewired 1000 times and an average of 100 networks was used.

### Step 4. Network Alignment between Subjects

Network alignment was performed using a matrix alignment algorithm implemented as part of the Brain Connectivity Toolbox [18]. This function uses simulated annealing to align two adjacency matrices relative to one another by reordering nodes in one of the matrices. At each iteration, the distance metric between the two matrices (in this case, the absolute difference) is calculated and minimized. Connectivity matrices must have the same number of nodes. In general, the outcome depends on the initial condition (the setting of the random number seed). Good solutions can be obtained for matrices up to about 100 nodes. If the two matrices are related, it is recommended to pre-align them, as was done automatically in our case due to similar head positioning in the scanner. The function was run 100 times and the solution with the lowest cost was selected.

To compare matrices, we calculated the Pearson correlation coefficient, similarly to *Cammoun et al.* [20]. For two matrices with the elements *x_ij_* and *y_ij_*, the Pearson correlation coefficient was calculated as
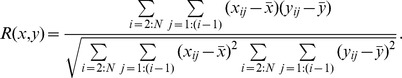
Because of the symmetry of the adjacency matrix, only the elements under the diagonal were considered (using a *for i = 2:N for j = 1:(i–1)* loop), resulting in a total of *N(N–1)/2* elements.

Network alignment was performed pair-wise between all subjects in each age group, between subjects from different groups (all combinations), as well as for the test-retest networks.

**Figure 3:**
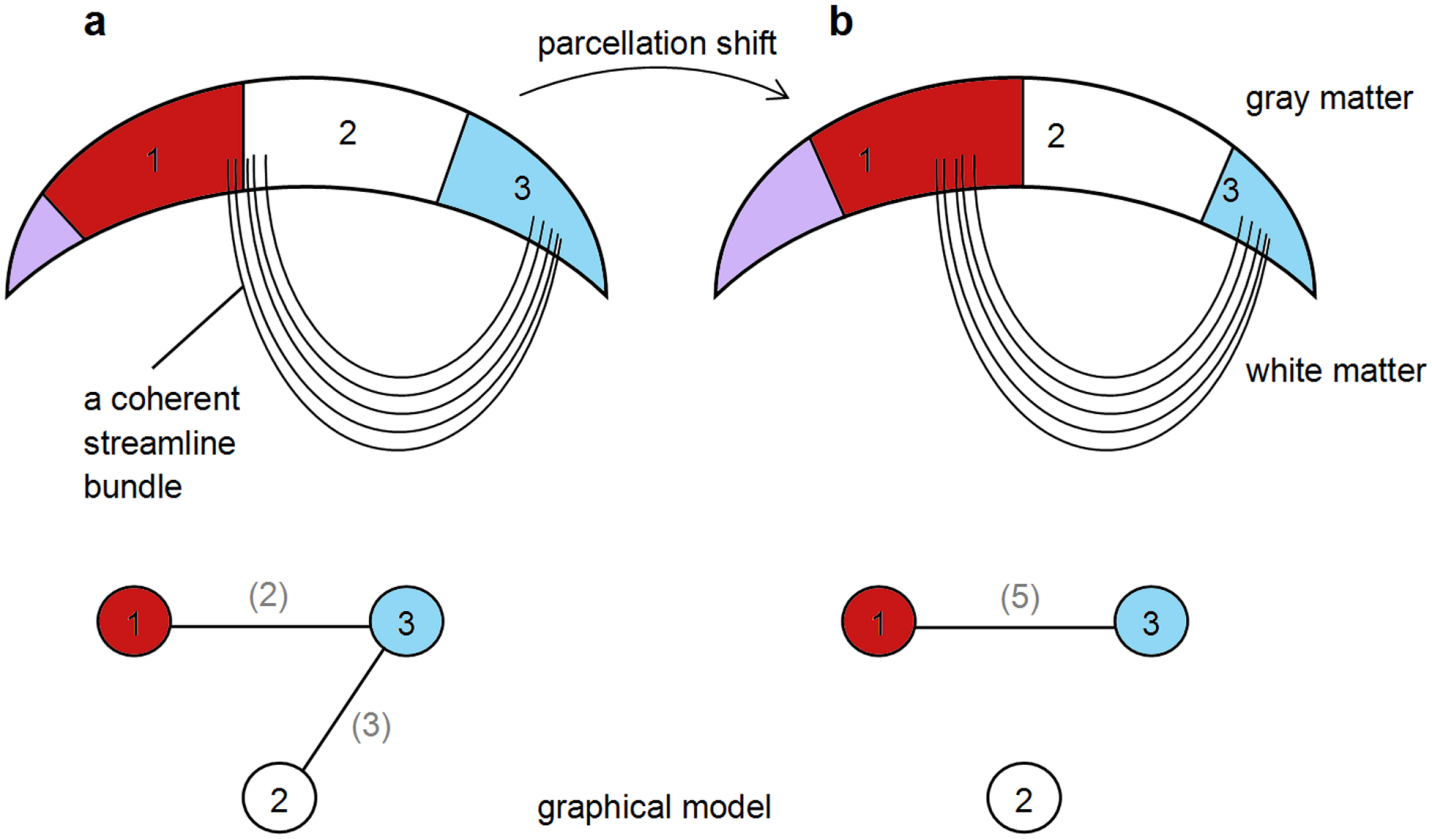
Schematic representation of the effect of shifting node boundaries: a) a coherent fiber (streamline) bundle falls on the border between two nodes, resulting in two binary connections to a third node; b) the bundle is contained within a node, resulting in only one binary connection.

To estimate the *error of node discretization*, the sphere used as the reference for the equal-area sphere partitioning was rotated around *x*, *y*, and *z* axes in steps that produce parcellation shifts of half of the node size. Network metrics were calculated and the rotated network was aligned with the initial, 0° network. The effect of *node discretization* is schematically illustrated in Figure 3. If a coherent fiber (streamline) bundle falls on the border between two nodes, this will result in two binary connections to a third node, whereas if the bundle is contained within a node, only one binary connection will be present. Whereas all brain networks in this study can be considered to be pre-aligned due to similar head positioning in the scanner, rotation to large degrees “destroys” the pre-alignment (making it similar to comparing two random networks), and the initial correlation coefficient is expected to be low.

### Step 5. Choosing the Cohort’s Reference Network

In order to bring all networks in a group of subjects to a common network space (and build a sum or average network), it is necessary to choose one network to which all others will be aligned. The alignment procedure is always performed between two networks, with specifying which network remains as it is (a reference network) and in which the nodes will be reordered. Although it is possible to pick the reference network randomly, this might result in the most deviant network of the cohort serving as the reference. To avoid this, we decided to determine the most typical network instead by performing pair-wise alignment of networks and calculating the Pearson correlation coefficient. The network with the highest average correlation coefficient was chosen as a reference network for the cohort.

### Step 6. Calculating the Average Brain Network and Edge-wise Analysis

Calculating the average brain network was performed by aligning all networks to the reference network and adding up the connections, which can be interpreted as creating a template in the network space.

This step can be followed by connection-wise comparison and statistical analysis to identify connections associated with a particular effect or contrast of interest, such as a group difference in a case-control comparison or a correlation with clinical measures. A t-test contrasting two groups is computed for each connection, based on the value stored in each subject’s connectivity matrix. Independent of the method that was used to map the connectome, the difficulty of this step is the inherent massive number of multiple comparisons that must be performed. For example, for 100 nodes it would be 4950 comparisons, for 95 nodes − 4465 comparisons, since an *N×N* adjacency matrix leads to a total of *N(N–1)/2* comparisons. To address this problem, two approaches are suggested: *i)* mass-univariate testing of the hypothesis followed by controlling the family-wise error rate (FWE) with a generic procedure such as the false discovery rate (FDR) [21]; or *ii)* the Network Based Statistics (NBS) for group comparison [22], which can be thought of as a translation of conventional cluster statistics [23] to a graph. The NBS can provide greater statistical power if the set of connections at which the null hypothesis is rejected constitutes a large component. The NBS comprises four steps:

1. Perform a two-sample t-test at each edge independently to test the hypothesis that the value of connectivity between the two populations stems from distributions with equal means.
2. Threshold the t-statistic available at each edge to form a set of suprathreshold edges.
3. Identify any components in the adjacency matrix defined by the set of suprathreshold edges (“observed components”). Compute the size of each observed component identified.
4. Repeat steps 1−3 *K* times, each time randomly permuting members of the two populations and storing the size of the largest component identified for each permutation. This yields an empirical estimate of the null distribution of the maximal component size. A corrected *p*-value for each observed component is then calculated using this null distribution.

It has to be noted that the NBS is of no use if the contrast does not form a connected component. However, if it does, the NBS has a greater utility as demonstrated, e.g., for the resting-state functional MRI data from patients with schizophrenia [22].

## Results

### Step 1. Defining all-to-all Cortical Connections

Figure 2 illustrates examples of tractograms for the three age groups. The resulting dense connectomes are not shown for the reason of their excessive size.

### Step 2. Parcellation of the Cortical Surface into N Equal-area Nodes

**Figure 4:**
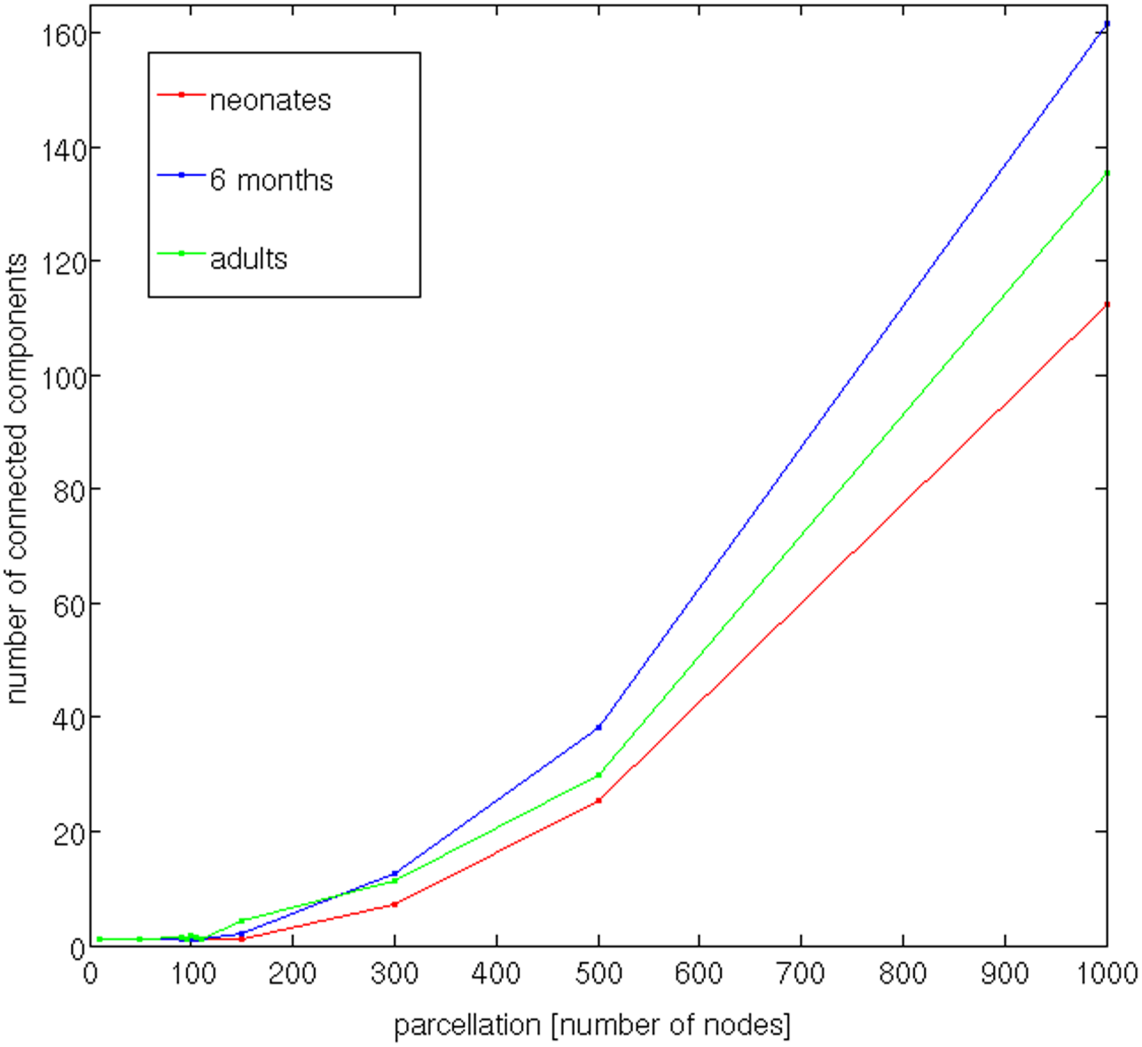
Dependence of the number of connected components on the parcellation.

With the increasing number of nodes (*N* = 10, 50, 90, 95, 100, 105, 110, 150, 300, 500, 1000, and 3000 nodes), the giant component was covering a smaller and smaller percentage of the brain cortex. Figure 2 illustrates the giant component mapped on the brain. Extended green areas indicate parts of the brain not covered by the giant component. Figure 4 shows the dependence of the size of the giant component (averaged across subjects) on the parcellation size for the three age groups, up to 1000 nodes. The optimal parcellation was determined at the highest node number value at which the number of connected components is still 1 for all subjects. At *N* = 95 all subjects had just one giant component covering the whole brain. Therefore, the number of nodes was set to 95.

### Step 3. Calculation of the 95×95 Network and Global Network Properties

**Figure 5:**
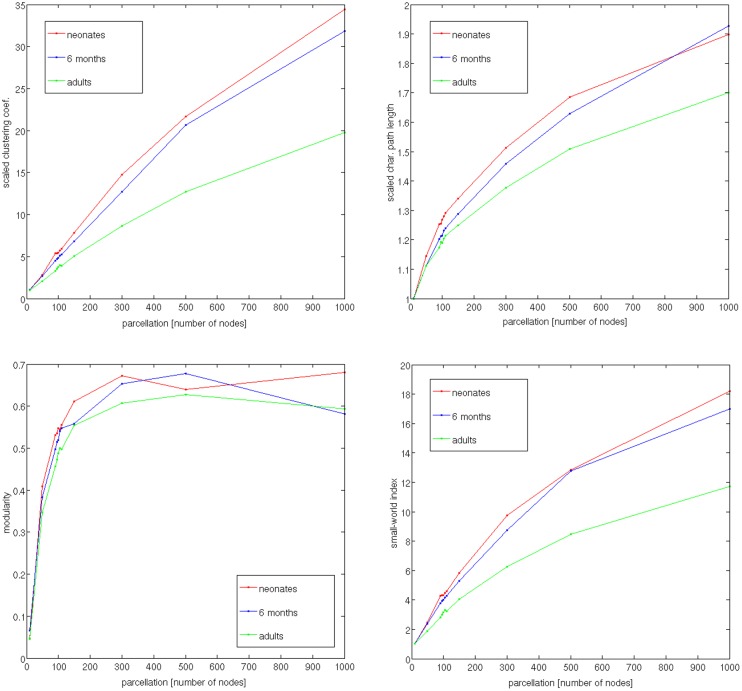
Dependence of the network metrics, averaged across subjects, on the parcellation: scaled (normalized) clustering coefficient *Cr*, scaled characteristic path length *Lr*, maximized modularity *Q*, and small-world index *swi.*

Figure 5 shows dependence of the network metrics, averaged across group’s subjects, on the parcellation: scaled (normalized) clustering coefficient *Cr*, scaled characteristic path length *Lr*, maximized modularity *Q*, and small-world index *swi.*

### Step 4. Network Alignment

**Figure 6:**
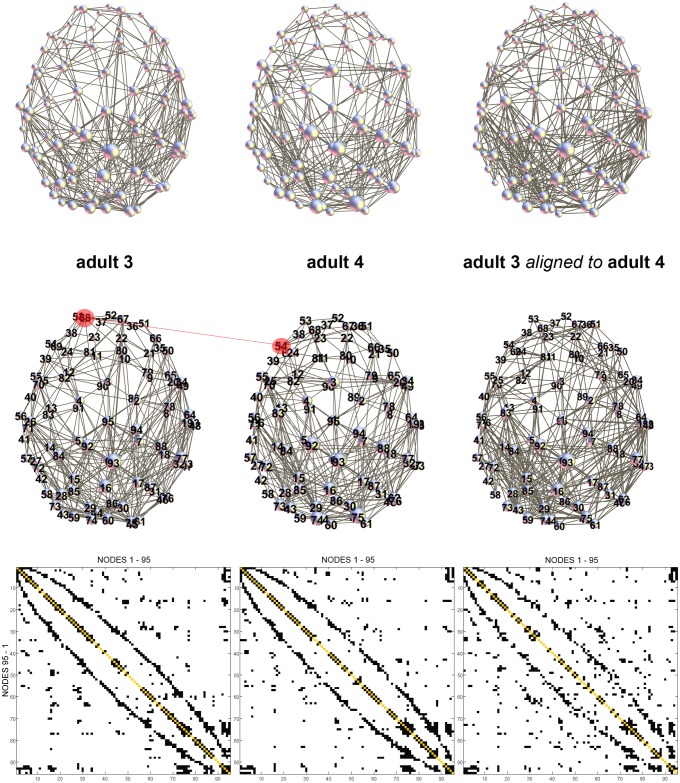
Example of network alignment for two adult brains (adult 3 and adult 4). Before alignment: *R* = 0.6019. After alignment: *R* = 0.6481. Many nodes of the adjacency matrix representing adult 3 stayed in the same location after reordering (see Table 1 for reordered indices). However, some nodes switched positions with its neighbors, e.g., as highlighted in red color: node 68 in adult 3 was aligned with node 54 in adult 4.

Figure 6 demonstrates an example of network alignment for two adult brains (adult 3 and adult 4). In this example, the correlation coefficient before alignment was 0.6019 and it increased after alignment to 0.6481. While many nodes of the adjacency matrix representing adult 3 stayed in the same location after reordering (see Table 1 for reordered indices), some nodes switched positions with its neighbors. An example is highlighted in red color in Figure 6: node 68 in adult 3 was aligned with node 54 in adult 4 (Table 1). The correlation coefficients after pair-wise network alignment were the following: for adults, mean = 0.6299, std = 0.0154, and range 0.6102–0.6410; for the 6-month-old infants: mean = 0.6322, std = 0.0101, and range 0.6213–0.6441; and for the neonates: mean = 0.6585, std = 0.0089, and range 0.6438–0.6673, with very low *p*-values (*p*≪10e-5). Tables 2−4 show the matrices of the correlation coefficients. The results of pair-wise comparison between subjects from different age groups are presented in Tables 5–7. The correlation coefficients between subjects from the adult and 6-month-old groups were, after alignment: mean 0.6093, std 0.0044, and range 0.5860–0.6294. Between the subjects from the adult and neonate groups we found the following: mean 0.5995, std 0.0047, and range 0.5813–0.6240. And between the 6-month-old and neonate groups we found these results: mean 0.6112, std 0.0037, and range 0.5780–0.6275.

**Table 1:** Reordered indices of the adjacency matrix of the adult 3 aligned to the matrix of the adult 4– see Fig. 6.

1	2	3	4	5	6	7	8	9	10	11	12	13	14	15	16	17	18	19	20
*7*	*2*	*3*	*4*	*5*	*6*	*34*	*20*	*9*	*22*	*11*	*12*	*13*	*14*	*26*	*16*	*48*	*33*	*19*	*8*
21	22	23	24	25	26	27	28	29	30	31	32	33	34	35	36	37	38	39	40
*10*	*21*	*36*	*24*	*25*	*40*	*41*	*15*	*29*	*30*	*32*	*31*	*49*	*18*	*62*	*52*	*37*	*53*	*69*	*39*
41	42	43	44	45	46	47	48	49	50	51	52	53	**54**	55	56	57	58	59	60
*56*	*27*	*71*	*59*	*44*	*47*	*64*	*17*	*54*	*61*	*67*	*51*	*23*	***68***	*55*	*70*	*57*	*42*	*60*	*43*
61	62	63	64	65	66	67	68	69	70	71	72	73	74	75	76	77	78	79	80
*45*	*46*	*63*	*88*	*38*	*66*	*50*	*80*	*81*	*83*	*58*	*72*	*95*	*84*	*74*	*76*	*77*	*78*	*65*	*79*
81	82	83	84	85	86	87	88	89	90	91	92	93	94	95	**ad4**				
*82*	*90*	*28*	*73*	*85*	*86*	*87*	*75*	*89*	*35*	*91*	*92*	*93*	*94*	*1*	***ad3***				

**Table 2:** Correlation coefficients after alignment of the adult brain networks. Adult 4 was chosen as the “reference brain” based on the highest correlation coefficients.

	adult 1	adult 2	adult 3	adult 4
adult 1	1.0000	0.6221	0.6158	**0.6102**
adult 2	0.6195	1.0000	0.6290	**0.6410**
adult 3	0.6183	0.6368	1.0000	**0.6507**
**adult 4**	**0.6102**	**0.6410**	**0.6481**	1.0000

**Table 3:** Correlation coefficients after alignment of the 6-month-old brain networks. Subject 1 was chosen as the “reference brain” based on the highest correlation coefficients.

	6mo 1	6mo 2	6mo 3	6mo 4
**6mo 1**	1.0000	**0.6277**	**0.6423**	**0.6441**
6mo 2	**0.6277**	1.0000	0.6217	0.6213
6mo 3	**0.6391**	0.6184	1.0000	0.6359
6mo 4	**0.6441**	0.6213	0.6359	1.0000

**Table 4:** Correlation coefficients after alignment of the neonate brain networks. Subject 3 was chosen as the “reference brain” based on the highest correlation coefficients.

	neo 1	neo 2	neo 3	neo 4
neo 1	1.0000	0.6438	**0.6555**	0.6557
neo 2	0.6438	1.0000	**0.6673**	0.6619
**neo 3**	**0.6555**	**0.6673**	1.0000	**0.6669**
neo 4	0.6557	0.6619	**0.6669**	1.0000

**Table 5:** Correlation coefficients after pair-wise alignment between subjects from the adult and 6-month-old groups.

	adult 1	adult 2	adult 3	adult 4
6mo 1	0.6145	0.5971	0.6056	0.6057
6mo 2	0.6323	0.5985	0.6067	0.6225
6mo 3	0.5902	0.5889	0.6117	0.6176
6mo 4	0.6175	0.6074	0.6277	0.6048

**Table 6:** Correlation coefficients after pair-wise alignment between subjects from the adult and neonate groups.

	adult 1	adult 2	adult 3	adult 4
neo 1	0.6080	0.5917	0.6186	0.6062
neo 2	0.6075	0.6002	0.6240	0.5980
neo 3	0.5888	0.5924	0.5907	0.5918
neo 4	0.5813	0.5857	0.6122	0.5954

**Table 7:** Correlation coefficients after pair-wise alignment between subjects from the 6-month-old and neonate groups.

	6mo 1	6mo 2	6mo 3	6mo 4
neo 1	0.6068	0.6036	0.6048	0.6236
neo 2	0.6024	0.5780	0.6098	0.6023
neo 3	0.6101	0.5982	0.6275	0.6232
neo 4	0.6226	0.6194	0.6270	0.6200

**Figure 7:**
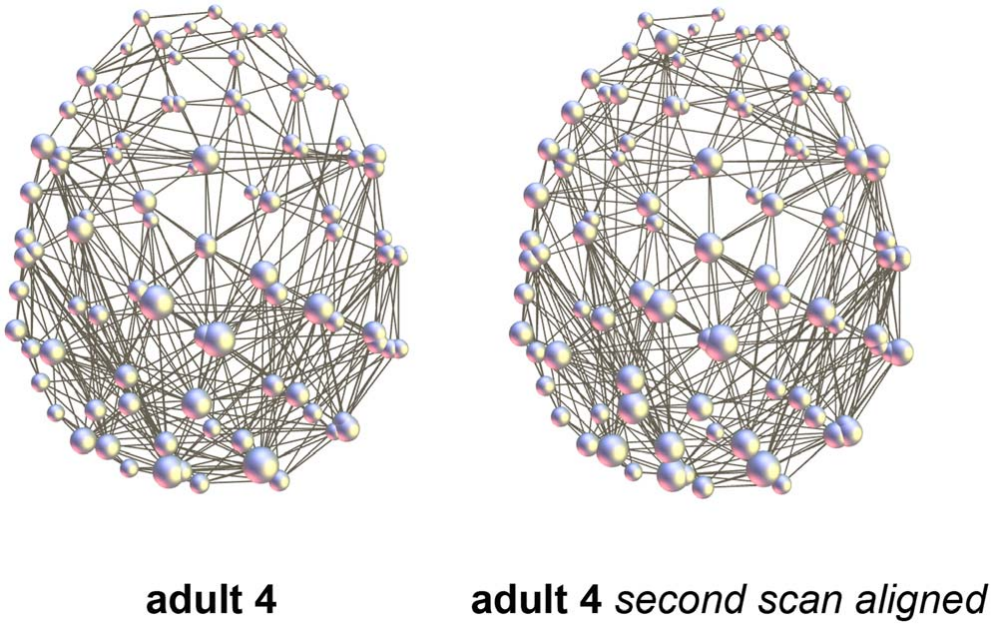
Test-retest example (adult 4). The second scan was performed minutes apart with a slight head displacement: *R* = 0.7037 after alignment.

Correlation coefficients for the aligned test-retest networks in the 6-month-old subject and adult were 0.7443 and 0.7037, respectively (Fig. 7). Statistically these values were significantly higher than inter-subject values in the corresponding group (*p* = 1.23e-6 and *p* = 7.88e-5, respectively).

**Figure 8:**
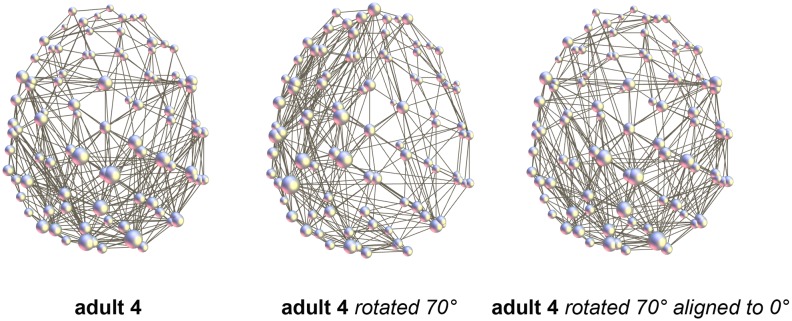
Rotation of the parcellation (adult 4). Example for the rotation of 70°_z_. *R* before alignment 0.4742, after alignment 0.7828. In the middle, the rotated network plotted onto the non-rotated anatomy is shown to illustrate the 70°_z_ rotation.

The results of the rotation of the parcellation are shown in Table 8. In order to produce parcellation shifts of half of the node size, the reference sphere was rotated 10° in both directions around the three axes, *x*, *y*, and *z*. In some cases the component covering the whole brain disconnected and network alignment could not be considered as properly performed. Alignment of the rotated networks with the initial, 0° network resulted in correlation coefficients of about 0.7, similar to the test-retest result. An example of alignment for a rotation of 70°_z_ is shown in Fig. 8 as an illustration. In this case, the correlation coefficient before alignment was 0.4742 and after alignment 0.7828.

**Table 8:** Rotation of the parcellation (adult 4).

rotation	0°	10°_x_	−10°_x_	10°_y_	−10°_y_	10°_z_	−10°_z_
total number of binary connections	832	818	820	750	792	746	802
number of connected components	1	**3**	**2**	1	1	1	1
*Cr*	5.71	3.29	3.25	3.73	3.24	3.58	3.55
*Lr*	1.18	1.19	1.21	1.21	1.19	1.20	1.18
*Q*	0.33	0.45	0.48	0.48	0.48	0.49	0.48
correlation coefficient	1	(0.6755)	(0.7039)	0.6891	0.7022	0.7139	0.7186

### Step 5. Choosing the Cohort’s Reference Network

Tables 2–4 show the results of choosing the cohort’s reference brain network. The network with the highest average correlation coefficient was chosen as the reference network of the cohort (marked in bold).

### Step 6. Calculating the Average Brain Network and Edge-wise Analysis

**Figure 9:**
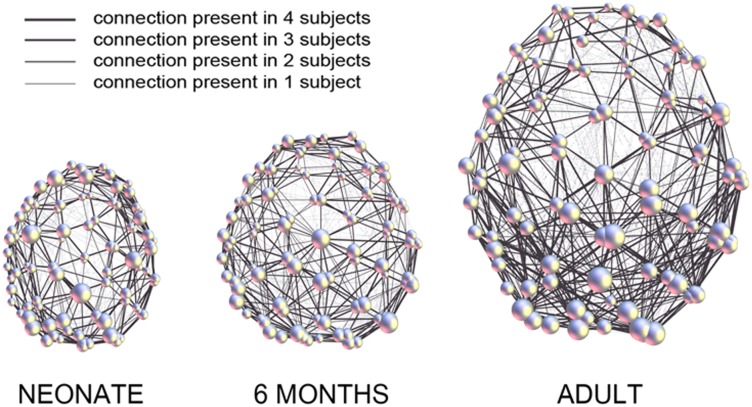
2D representation of atlas-free brain networks with *N* = 95 equal-area nodes, showing average structural connectivity in three age groups: neonates (4 subjects), 6-month-old infants (4 subjects), and adults (4 subjects). The sum of connections in all subjects of each group (*n* = 4) was calculated after alignment of their networks to the respective reference network and mapped onto the anatomy of the “reference brain.” Thicker lines represent connections present in more subjects. Bigger spheres represent nodes of higher degree.

Figure 9 demonstrates the average brain networks for the three age groups (with *n* = 4 subjects in each group), mapped onto the anatomy of the corresponding “reference brain.” Thicker lines represent connections present in more subjects.

## Discussion

The view of the brain according to which cognitive functions are localizable, known as *localism*, as well as the view that the entire brain participates in each cognitive function, known as *holism*, both contributed to connectomics, which views the brain as a network of interacting brain regions. It should be emphasized that brain is organized as a network on multiple scales: synaptic connections between single cells, connections among cell populations within individual anatomical regions, and finally the large-scale architecture of brain regions and their interconnected pathways [24]. Different scales of brain organization offer parallel and complementary views and brain connectivity at the large scale mapped using MRI should not be regarded as a poorly resolved approximation of the underlying microscopic order [24]. The long-term goal of MRI connectomics should be seen in advancing neuroscience and medical research by identifying the network substrate of normal and impaired cognition and behavior, as well as of normal and abnormal development. Acquisition of this knowledge would aid diagnosis, treatment monitoring, and outcome prediction for neurologic and psychiatric disorders.

### Why not use More Standardized Approaches Based on Brain Atlases?

Anatomical co-registration between brains and atlas-based parcellation into nodes have allowed for exploration of human brain networks and provided insights into network disruption in different neurologic and psychiatric disorders [4]. However, anatomical, cytoarchitectural, and functional variability is observed even in healthy individuals, especially in higher order association cortex areas [25], [26]. The impression of a match between sulcal landmarks and areal borders may lead to the wrong conclusion that landmarks are sufficient for the localization of a cytoarchitectonic border [6]. Maturation and reorganization processes can lead to an even more dramatic bias towards the standardized adult brain anatomy. These problems motivated us to develop the “brain without anatomy” approach, which uses network architecture to co-register and compare brains. Rationale for our approach lies in the fact that while cytoarchitecture influences the brain’s connective architecture, there is strong evidence to indicate that the reverse is also true, such as formation of novel cytoarchitectonic regions after artificially modifying cortical projections [15]. There is also strong evidence that connectivity has a profound influence in the determination of functional specificity within the cortex and attempts have been made to perform connectivity-based parcellation of the human cortex using diffusion MRI and tractography (see [14] for a review). A further interesting argument can be derived from a recent study by *Raj & Chen* who, using MRI connectomics, provided supportive evidence for the claim that economical wiring is a driving principle of the brain and that *connectivity determines anatomy* [27]. The authors performed two sets of analyses, in one of which they found that the connectivity structure of the brain has near optimal (cheapest) wiring cost compared to random networks with the same number of edges, degree distribution, and edge weight distribution. No cheaper wiring could be found without significantly degrading network performance. In another set of analyses, they kept the observed brain network topology and connectivity but allowed nodes to freely move on a 3D manifold topologically identical to the brain in order to find lowest wiring cost configuration. This resulted in a configuration with a striking resemblance to the brain.

In the present study, we propose a framework for the construction and connection-wise comparison of fully network-driven large-scale structural connectomes that is not constrained by anatomy. The assumption that was made in this study is that as long as there are certain cortical areas interconnected in a way that is efficient or fault-tolerant in terms of its network properties, it does not matter *where* those cortical areas are located anatomically or *how* they are connected in terms of the shape of the fiber bundle. It is assumed that, if the brain managed to reorganize itself after injury or surgery in a way that approaches the “normal” network organization, a respective recovery of its function can also be expected. Similarly, complete reversal of the right-left sides of the brain network would not result in differences between anatomy-free networks, because the network alignment process would flip the two sides of the network (which might be further explored as a method of correcting for brain lateralization).

To illustrate the approach, we applied the framework to three narrow age groups of the same gender: neonates, 6-month-old infants, and adults. Based on the calculated pair-wise correlation between subjects within each group (Tables 2–4), we observed that the 6-month-old infants were slightly more similar to each other after alignment (higher coefficients) than the adults. The neonates showed the highest similarity within the group. Pair-wise correlation between individual subjects from different groups was lower than within groups, with the lowest correlation between adults and neonates (Tables 5–7). The number of subjects used in this study to illustrate the approach was too small for an edge-wise group comparison.

### Technical Considerations

#### To weight or not to weight?

Various approaches to weighting network connection have been used in other studies, streamline count being the most common [17]. However, to date, no index derived from tractography has been proposed that can provide a physiologically meaningful quantification of the connection strength [17]. One of the problems associated with using streamline count as weights in developmental studies is that smaller brain sizes will lead to fewer streamlines per parcellation. This problem is compounded by other methodological biases related to brain volume and diffusion anisotropy changes; specifically, tractography studies do not register the higher number of axonal branches present in early development and instead show a greater number of streamlines with increasing age [28]. To overcome this limitation, we take into consideration all edges that are composed of at least one streamline. This approach can be considered as applying a fixed threshold of 1 streamline, thereby obviating the problem that smaller brains will have fewer voxels and fewer streamlines per parcellation. Interestingly, while the total number of streamlines between the studied age groups varied significantly (up to 5 times as many streamlines in the adults compared to the neonates after applying the length threshold), the highest *N* at which all nodes were still interconnected (the optimal parcellation) and the number of non-zero entries (*nnz*) at *N* = 95 nodes varied much less across the age groups: for the adults *nnz* = [898; 812; 876; 832], for the 6-month-olds *nnz* = [706; 666; 646; 676], and for the neonates *nnz* = [682; 718; 668; 640]. One might still argue that streamline count can be utilized in network alignment to avoid complete “flatness” of the networks, whereby an edge consisting of only one streamline (arguable less reliable) plays the same role in alignment as an edge comprised by many streamlines. However, a varying total number of streamlines and a highly uneven distribution of streamline counts across subjects, even within a group, have led to meaningless results of the network alignment algorithm (results not shown). Sampling different metrics of white matter microstructure (myelination, axon density, axon diameter) along streamlines, which is the goal of a new comprehensive method called *tractometry* [29], might provide a biologically more accurate quantification of connectivity and a more meaningful weighting scheme.

#### How many nodes?

As the first step of the framework, our approach uses atlas-free brain parcellation with an optimal choice of node size and number for a given population, acquisition and tractography parameters [11]. The method utilizes equal-area node partitioning and does not rely on any anatomic atlases or landmarks. The optimal number of nodes is directly derived from the data. The only assumption made is that no regions of the brain are structurally isolated from the rest of the brain. The interconnectedness of all nodes is also a prerequisite for the subsequent network alignment, as is the equal number of nodes among subjects. The highest number of nodes at which this condition was fulfilled in all studied subjects was 95. The number of nodes will generally depend on the acquisition and tractography parameters; the relatively low number of nodes we obtained (and, arguably, a relatively coarse resulting parcellation) can be increased, for example, by using diffusion spectrum imaging (DSI) [30]. *Bassett et al.* showed that, while reproducibility of network properties was higher in DTI, DSI networks were characterized by an increased number of reconstructed tracts [31]. However, different network alignment algorithms would be required, because with the use of the matrix alignment algorithm with simulated annealing from the Brain Connectivity Toolbox used in this study, good solutions can be obtained only for matrices up to about 100 nodes (http://www.brain-connectivity-toolbox.net). In the case of standard methods involving anatomical co-registration, finer parcellations were shown to lead to an increasing impact of measurement and processing noise [14], [20], [31], as will be further discussed in the following section on precision. Therefore, a potential problem of high-resolution atlas-based connectome analyses is that the noise inherent to the mapping procedure might overshadow group differences [20]. A possible explanation suggested by *de Reus & van den Heuvel* [14] is that with smaller brain regions, the total number and area of regional boundaries become larger. As a result, variation in the location of region boundaries, caused either by registration difficulties or biological differences, is more likely to change the assigned source and target region of a reconstructed white matter fiber tract [14].

We also observed a strong influence of the number of nodes on the values of global network properties (Fig. 5), as shown for other parcellation schemes (see [14] for a review). This should always be kept in mind when comparing network properties resulting from studies that utilize different parcellation schemes and scales. With the parcellation into 95 nodes, as well as with the majority of the studied values of N, the average global network properties of the three age groups were in agreement with the previously reported trend to increasing integration and decreasing seggregation with age [10], [32]–[34]. Namely, the scaled characteristic path length and the average clustering coefficient both decreased with age (Fig. 5). The observed increases in network integration can be explained by the strengthening of axonal projections, particularly longer range association projections, during the first years of life [35]. The small-world index was observed to decrease with age, mediated by a stronger decrease of the clustering coefficient than characteristic path length.

#### Precision

The method’s reproducibility was estimated by test-retest scans in one subject from the 6-month-old group and one adult, resulting in correlation coefficients of 0.7443 and 0.7037, respectively. While statistically significantly higher than inter-subject values, these relatively low test-retest values can arguably limit the method’s capability of detecting group differences. The relatively large size of the nodes arbitrarily (“randomly”) positioned on the cortex largely explained the variability, as was revealed by rotating the parcellation (Fig. 3 and Fig. 8), although measurement noise is assumed to play an additional role. In a study of brain connectomes mapped at different scales, *Cammoun et al.* [20] observed highest test-retest correlation coefficients for resolutions of 133 and 241 nodes. They showed that high-resolution parcellations lead to higher (anatomical) registration errors. As already mentioned above, all types of variation, either resulting from reprocessing scans, repeatedly scanning the same subject or comparing different subjects, were found to increase with increasing the number of nodes [20]. Using weighted matrices, *Cammoun et al.* obtained test-retest values ranging from 0.874 to 0.976 [20]. However, one should not compare directly correlation coefficients for weighted and binary matrices: the correlation coefficients for our binary matrices were 0.7443 and 0.7037, but these values increased to 0.8967 and 0.8618, respectively, when using the original streamline count as weights.

#### Accuracy

It is important to remember that DTI is a rather approximate technique and that diffusion MRI measures only the dephasing of spins of protons in the presence of spatially-varying magnetic fields [17]. Nevertheless, MRI tractography is, to date, the only method that permits the calculation and visualization of fiber tract trajectories in optically turbid tissue *in vivo* and was developed, in part, to address this unmet need [36]. The accuracy of MRI tractography can only be tested by comparison with human histology (although the sample dissection, freezing, dehydration, fixation, microtoming, thawing, etc. can alter tissue microstructure) and invasive tract tracings in non-human primates. However, even invasive tracer methods, which are considered to be the most reliable way to study connections in real brain tissues and serve as the gold-standard, suffer from methodological problems that might cause disparities when comparing them with MRI tractography [37]. The approach proposed in this paper will benefit from any future developments and improvements in tractography methodology that lead to a higher accuracy of tract reconstruction.

It should be noted that the step of extraction of the subcortical surface (*Step 1, iii)*) was performed using the diffusion dataset and not a high-resolution anatomic image as it is commonly done. Consequently, no co-registration of brain scans in the anatomic domain was performed. On one hand, availability of such additional images is not required; on the other hand, the diffusion dataset has a low resolution and after additional smoothing provides only an approximate representation of the subcortical surface. This choice, however, is consistent with the different strategy presented here, in which no registration with standard brains is performed and accurate anatomical location of nodes is deemphasized. Connections between nodes are determined by whether or not streamlines *intersect* the respective patches of the subcortical surface.

In addition to the mentioned accuracy issues, the question of validation is particularly tricky for networks themselves. Although graph properties are commonly used to characterize the brain’s organization, the link between these properties and the brain’s ability to segregate, integrate, modularize, process or transmit information is completely unknown [19]. We expect, however, that increasing experience using the rapidly developing field of MRI connectomics in combination with the well-established field of graph analysis will narrow the existing gap between our understanding of brain network topology on one side and of human behavior and cognition on the other side [4].

In summary, this study describes a method for atlas-free construction and connection-wise comparison of diffusion MRI-based brain networks. This current work represents the extension of the previously proposed template-free parcellation [9]–[11] by introducing the novel idea of using network alignment to replace the morphology-based alignment of individual brains used in most structural connectivity studies. Through the abstraction from the anatomy, the framework developed in the present study allows for a fully network-driven analysis of structural MRI connectomes and can be applied to subjects at any stage of development with any potential anatomical abnormalities. After networks have been aligned and the mean group network created, groups can be compared using existing frameworks, such as the Network Based Statistics [22]. Alternatively, comparison can be performed by means of other statistical testing or classification approaches [19]. Thus, the framework enables comparison of both global and local network properties of any two brain networks. When global network measures, such as clustering coefficient or characteristic path length are used, little insight can be gained about the details of potential plasticity effects, maturational or pathological processes, as local phenomena get diluted in the global mean [19]. Therefore, node-wise and edge-wise comparison is important. The proposed framework has been illustrated in relatively homogeneous samples from three narrow age groups of the same gender. Future research directions might include further exploration of the “brain without anatomy” approach with other alignment algorithms (e.g. with flexibility with respect to the number of nodes in order to allow for emergence of new nodes), inclusion of subcortical structures (e.g. thalamus), or use of functional networks. The present study is intended to stimulate further developments of this new, fully network-driven way of looking at the brain and comparing brains of different subjects.
